# Smart sensor tights: Movement tracking of the lower limbs in football

**DOI:** 10.1017/wtc.2021.16

**Published:** 2021-11-29

**Authors:** Annemarijn Steijlen, Bastiaan Burgers, Erik Wilmes, Jeroen Bastemeijer, Bram Bastiaansen, Patrick French, Andre Bossche, Kaspar Jansen

**Affiliations:** 1 Faculty of Electrical Engineering, Mathematics and Computer Science, Delft University of Technology, Delft, The Netherlands; 2 Faculty of Behavioral and Movement Sciences, Vrije Universiteit Amsterdam, Amsterdam, The Netherlands; 3 Centre for Human Movement Sciences, University Medical Centre Groningen & University of Groningen, Groningen, The Netherlands; 4 Faculty of Industrial Design Engineering, Delft University of Technology, Delft, The Netherlands

**Keywords:** inertial measurement units, wearable sensors, football, movement tracking

## Abstract

This article presents a novel smart sensor garment with integrated miniaturized inertial measurements units (IMUs) that can be used to monitor lower body kinematics during daily training activities, without the need of extensive technical assistance throughout the measurements. The smart sensor tights enclose five ultra-light sensor modules that measure linear accelerations, angular velocities, and the earth magnetic field in three directions. The modules are located at the pelvis, thighs, and shanks. The garment enables continuous measurement in the field at high sample rates (250 Hz) and the sensors have a large measurement range (32 g, 4,000°/s). They are read out by a central processing unit through an SPI bus, and connected to a centralized battery in the waistband. A fully functioning prototype was built to perform validation studies in a lab setting and in a field setting. In the lab validation study, the IMU data (converted to limb orientation data) were compared with the kinematic data of an optoelectronic measurement system and good validity (CMCs >0.8) was shown. In the field tests, participants experienced the tights as comfortable to wear and they did not feel restricted in their movements. These results show the potential of using the smart sensor tights on a regular base to derive lower limb kinematics in the field.

## Introduction

Over the years, the physical demands of football have increased. In the English Premier league for example, high intensity running distance and sprinting distance increased by more than 30% between 2006 and 2012 (Barnes et al., 2014). Likewise, in World Cup Soccer finals between 1966 and 2010, ball speed and passing rate increased by 15 and 35%, respectively (Wallace and Norton, 2014). The high physical demands in football lead to a substantial injury risk. In professional football 8.1 injuries per 1,000 hr of play were reported based on epidemiological data of 44 studies (López-Valenciano et al., 2020). Most injuries occur during competitive matches (Owoeye et al., 2020), and almost one-third of all time-loss injuries are muscle related. More than 90% of all muscle injuries are lower limb injuries, of which 37% concern the hamstrings (Ekstrand et al., 2011). Several studies conclude that increased age and previous injury significantly increase the risk of hamstring injury (Arnason et al., 2004; Freckleton and Pizzari, 2013). Moreover, Ekstrand et al. (2011) reported more cases of injury at the end of each half of a football game, which suggests that fatigue is also an important risk factor. Therefore, investigating physical player load and its relation to hamstring injury prevalence can expand the knowledge on the etiology of hamstring injuries, and help develop new methods to prevent these injuries in the future.

The use of wearable electronic measurement equipment was approved by the FIFA in 2015 (Dunn et al., 2018). Several commercial systems that can track individual athletes by GPS (Global Positioning System, e.g., Zephyr Performance Systems, US, Catapult Sports Ltd., Australia and JOHAN Sports, The Netherlands) or RFID technology (Radio Frequency Identification, e.g., Inmotio Object Tracking, The Netherlands) have been developed. In addition, the systems contain a 3-axis accelerometer. Based on the data from these systems, the total distance covered can be calculated, whole-body acceleration and deceleration data can be derived (Barrett et al., 2014) and activities can be classified (Datson et al., 2017). However, these data do not provide insight in the kinematics of the lower limbs. For detailed kinematic analysis of the lower limbs, the current practice is to use an optoelectronic measurement system (Cuesta-Vargas et al., 2010; Schache et al., 2012; Malfait et al., 2016), which is restricted to a lab environment and does not allow for on-field measurements during training or competition. Kinematic measurements outside the lab are commonly performed using inertial measurement units (IMUs), which are attached to body segments. IMUs measure linear accelerations (accelerometer), angular velocities (gyroscope) and the earth magnetic field (magnetometer) in three different axes making the nine degrees of freedom (Ahmad et al., 2013). The orientation of an IMU can be derived by combining these measurements using a sensor fusion algorithm. After a calibration of the sensors to each limb segment, the lower limb kinematics can be obtained (Luinge et al., 2007; Roetenberg et al., 2007).

Recently, several applications of IMU systems have been presented. These IMUs consist of a box, typically with a size of 30 mm × 40 mm × 10 mm, containing all electronics including the battery. In most cases, the modules are tied around the limbs, chest, or hands (Stiefmeier et al., 2008; Rawashdeh et al., 2016; Anwary et al., 2018; Chen et al., 2020; Teague et al., 2020). Other researchers attach them to the skin with an adhesive at predetermined locations (Gaidhani et al., 2017; Hu et al., 2020). These sensor units are less suitable for long-term monitoring studies and little attention is paid to the integration of the IMUs in clothing in these papers. The company Xsens developed a motion tracking suit including software to derive body kinematics (MVN Link, Xsens, The Netherlands). Although, they found an elegant way to place the hardware in pockets in the garment, the electronics are relatively large, and it takes time and additional help to position all the electronics in the right locations when wearing the suit. The integration of electronics in textile is part of another research field that is more focused on materials science (Varga, 2017; de Mulatier et al., 2018; Komolafe et al., 2019). In this field, some researchers have integrated IMUs in textiles. For example, Wicaksono et al. created a suit with an IMU on a flexible printed circuit board (PCB) right below the sternum for measuring heart, and breathing activity (Wicaksono et al., 2020), and Wang et al. (2015) created a garment with integrated IMUs for posture monitoring. However, in all of the above mentioned monitoring systems, either the sensor modules are relatively large and not integrated in the garment, or the IMU sampling rate and detection range are limited (100 Hz or below, and 16 g or lower, respectively). Therefore, as far as we are aware of, a garment for unobtrusive monitoring of lower limb kinematics in everyday training situations which is accurate enough and can be used without the need of technical assistance does not yet exist.

To enable reliable long-term monitoring at the football field, we are developing an easy-to-use wearable monitoring system with integrated IMUs, which will be referred to as smart sensor tights. This is an embedded system with IMUs integrated in textile on each segment of the lower limbs. The sensors are connected by flexible wiring to a central processing unit at the waist band that contains a single microprocessor and a power source. The advantages of this specific architecture are that the sensor modules are small, unobtrusive, and of low weight. Moreover, no additional synchronization of the sensors is needed, since they are all read out by one microprocessor. To the best of our knowledge, this is the first IMU system that has the sensors integrated in shorts or tights, which allows for easy on-field measurements of fast movements of the lower limbs. By making use of a real-time operating system, high sample rates can be reached to track fast football-specific movements accurately. The measurement range of the IMUs is larger (±4,000°/s for the gyroscopes and ±30 g for the accelerometers) than in the systems presented above. The tights can be easily put on by the players themselves and can be worn in regular matches and trainings. The integration of the sensors in a garment facilitates long-term monitoring of players and larger scale studies outside a lab environment, which are required to find injury risk factors for injury prevention.

This article presents the design and development of the smart sensor tights. First, the smart sensor tights system design is presented, followed by the detailed design of hardware and software. Secondly, a controlled test with football-specific movements was executed in the lab to concurrently validate the novel sensor tights with an optoelectronic measurement system. Third, a validation study with the complete system was executed. The sensor tights were worn during football-specific exercises to test the performance in the field. Lastly, user experience is assessed.

## Materials and Methods

To improve the technical reliability and user acceptance of the new system, an iterative design approach was followed. Multiple prototypes were made to enable simultaneous improvement of the hardware and software, as well as improvement of usability and comfort. Prototype one included widely used IMUs (MPU-9250, InvenSense, San Jose, CA) and an easy-to-use microcontroller development board (Arduino Due). With this prototype, a proof of concept was created and based on field tests, major improvements were identified. These improvements included increasing the sampling rates, increasing the measurement range, and improving the robustness of the electronics. This formed the starting point for the design of the second prototype that is presented here.

### Design

The design of the sensor tights had to meet several requirements. To investigate the kinematics of the legs, five IMUs are integrated in this design. IMUs are placed on the thighs, shanks, and pelvis. The exact locations are explained below. Since the product will be used by researchers in biomechanics to derive lower limb kinematics, it is important that the IMUs have a sample rate of 250 Hz in each direction and that raw data can be obtained via an SD card. At a later stage, a wireless connection will be integrated. Furthermore, the risk that the electronics harm the wearer or fellow players during a match should be negligible. The garment should not restrict the players movement and it should be comfortable to wear. Lastly, the IMUs need to be tight to the skin and of low mass, to prevent shaking due to inertia of the sensors.

#### Hardware design

**Figure 1.**
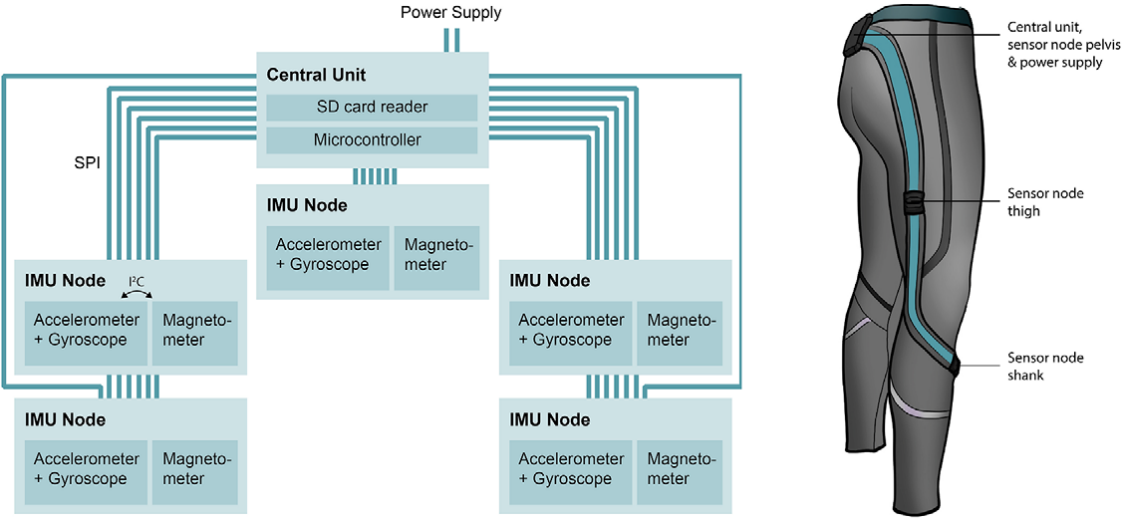
System overview and location of the electronics.

For the hardware, it was chosen to centralize the data acquisition electronics and the power supply. This resulted in tiny and low weight sensor modules that reduced the measurement artifacts due to inertia of the sensors. Figure 1 shows an overview of the system. The two subsystems are referred to as the IMU node and the central unit. A new PCB was designed for each subsystem. Linear accelerations and angular velocities are measured by the ICM-20649 (InvenSense, San Jose, CA). This IMU was selected based on the wide measurement range of ±4,000°/s and ± 30 g, which enables more representative analysis of kinematics of highly dynamic movements. Preliminary experiments showed that with sensors with a smaller measurement range (16 g), clipping occurred at moments of impact with the ground. Via an I^2^C bus, a magnetometer (AK8963) with a range of ±4,900 μT (Asahi Kasei Microdevices, Japan) is connected to the ICM-20649. The central unit is responsible for reading out the sensor, power handling and data storage at an SD card. The microcontroller that was chosen for the central PCB is the Arm Cortex -M4 with FPU processor (Arm Ltd., UK) which runs at 100 MHz maximum. The STM32F411 Nucleo-64 development board, was used for software development (STMicroelectronics, Switzerland). Lastly, a CE marked power bank (1,350 mAh, Xqisit, Germany) was used as a power supply. Data acquisition from the sensor nodes occurs via an SPI connection. The SPI protocol was chosen, because the ICM-20649 can then directly be read out through SPI and no extra electronics are needed. Furthermore, the SPI protocol is less prone to errors over long distances at high data transfer rates than the I^2^C protocol.

#### Sensor locations

Two requirements needed to be considered to identify the best locations for the sensors. First, soft tissue artifacts (STAs) need to be minimized (Barré et al., 2015). STAs are defined as measurement errors that arise from the relative motion between bone and sensor, for example due to muscle bulging during a contraction. Second, we needed to choose a location that is safe and comfortable for use in a football context. To investigate the best sensor locations based on comfort and safety, football training sessions were observed from the Dutch national football team under 21 and a survey was held among the team (Steijlen et al., 2020). In consultation with human movement scientists and physiotherapists, locations that were expected to introduce the least STAs were identified. Based on the outcomes, the upper leg sensors are placed halfway the hip and knee joints on the lateral side of the thighs. STAs were expected to be relatively low because of the stiff underlying connective tissue. The lower leg sensors are placed at the inner sides of the shanks, close to the knees. The trunk sensor is placed in the waistband and the central unit, including the battery, is placed in a pocket located above the sacrum (Figure 1).

#### Software design

To make sure that each sensor node can be read out with a sample rate of 250 Hz for the gyroscopes and accelerometers and 100 Hz for the magnetometers, a real-time operating system (FreeRTOS) was used for this design. An RTOS was used to implement a pre-emptive priority-based scheduling scheme (Guan et al., 2016). Pre-emptive scheduling means that a task with lower priority can be interrupted by a task with higher priority. Once the task with a higher priority is finished, it will continue with the lower priority task. Table 1 shows the different tasks priority level. With this software, the required sample rates can easily be reached. When the microcontroller is running at 100 MHz, it turns out that the microcontroller only needs 40% of available time to execute all tasks. The power consumption is around 110 mAh, which means that with the current battery the system can run for 12 hr.

**Table 1.** Scheduling scheme for microprocessor

Task	Frequency	Priority
Read out accelerometers and gyroscopes for all IMUs	250 Hz	Real-time
Store accelerometer, gyroscope, and magnetometer data into a buffer (size: 4 kb)	250 Hz	Real-time
Read out magnetometers for all IMUs	100 Hz	High
Create SD card file and handle the user interface	5 Hz	Above normal
Write the buffer to the SD card	Event-based	Normal

##### Lossless data compression

Future prototypes must be able to send data wirelessly to the football coach or medical staff. Given that the total amount of bits per sample is 16, the data rate of one prototype is 144 kbit/s. This implies that, when 22 players at the field are wearing the system, a data rate of 3.168 Mbit/s would be required. Based on the overview of different wireless protocols and their maximum bit rates and ranges by Kos et al. (2019), it can be concluded that only Wi-Fi protocols would be suitable. On top of that, experimental findings from literature show that the presence of a human body between the transmitter and receiver can lead to significant disturbances in the radiation pattern (Kurusingal et al., 2010; Sivaraman et al., 2010) To improve the reliability of wireless data transmission for future versions, the amount of data retrieved from the sensors can be compressed. Therefore, the FELACS data compression algorithm (Kolo et al., 2015) was implemented in the software. The performance of the algorithm was thoroughly tested in a football-specific setting.

### Prototype

**Figure 2.**
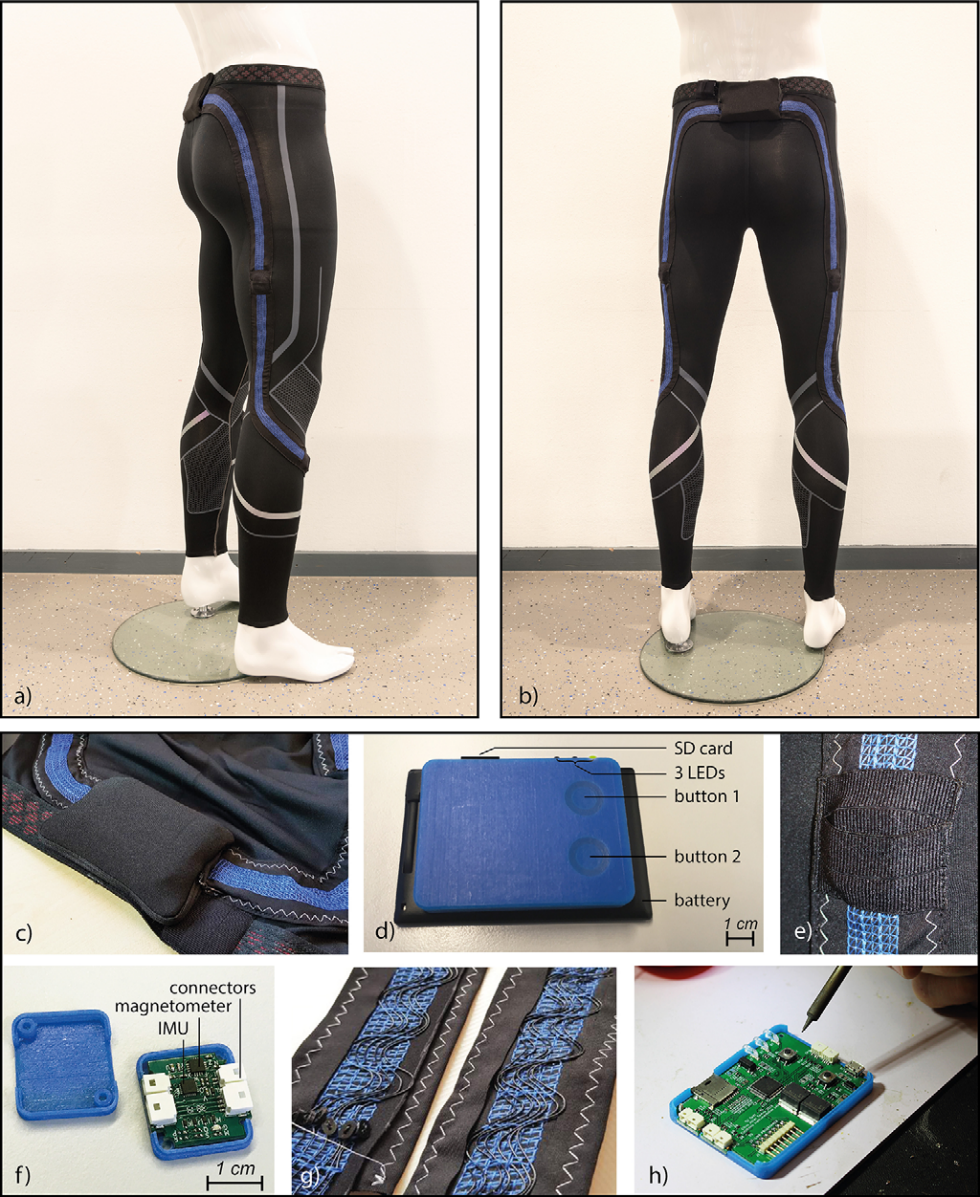
(a) Side view sensor tights, (b) Back view sensor tights, (c) Pocket with central unit PCB and battery, (d) Battery and central unit with user interface, (e) Pocket of a sensor node, (f) A sensor node, (g) Interlaced wiring that allows for 100% stretching, and (h) Central unit during assembling.

Figure 2 shows images of the tights and important details. As can be seen in Figure 2g an insulated stranded silver-plated copper conductor with a conductor area of 0.03 mm^2^ was chosen and laced in a serpentine pattern to allow for stretchability on top of a base garment (stretchable running tights, Under Armour, Dallas, TX). PTFE was chosen as insulation material, because of its high chemical inertness, hydrophobicity and mechanical strength, which will be of great value when the tights will absorb sweat and when they are washed. Miniature connectors for use in wearables are not yet commercialized. As an alternative, a regular miniature wire-to-board connector (Pico-clasp, Molex, Lisle, IL) was chosen to connect to the central unit PCB and IMU node PCBs. To test the impact of washing with soap and water on the reliability of the connections, a washing test (40°C, 1,200 RPM, including other sports clothes) was performed with these connectors (*n* = 12) and a 4-point resistance measurement was performed after each washing cycle. The resistance slightly increased after seven washing cycles. However, it stayed below 1 Ω. The PCBS were placed in 3D-printed casings made from a photopolymer (Connex 3, Objet 350, Stratasys Ltd., Israel). The sensor nodes weigh 3.6 g. The sensor nodes and central unit were placed in sleeves, which have a waterproof lining, to protect the electronics against sweat. For washing the tights, the sensors and central unit, can be disconnected. The wiring and connectors remain embedded in the garment. The central unit has an interface with three colored LEDs and two buttons, to start and stop recording and to indicate a special event. Figure 2d shows the interface.

### Validation Study

The validation of the design was split in two parts. The first part was a validation test in the lab. The aim of this test was to assess the concurrent validity of the prototype with the golden standard optoelectronic motion analysis system. The second part was a validation test of the system on the football field. In this experiment, the functioning of the device was tested, and user experience tests were performed. It was verified if the datasets are complete, and the performance of the data compression algorithm for different football-specific movements was measured. The study was approved by the Human Research Ethics Committee of Delft University of Technology. All participants gave informed consent.

#### Lab validation

As explained in the introduction, detailed lower limb kinematics can be derived by applying sensor fusion algorithms to the IMU data and performing sensor-to-segment calibration (Roetenberg et al., 2007). Recently, a paper has been published which explains the model that we use to derive lower limb kinematics with the sensor tights (Wilmes et al., 2020). In that study, individual sensor modules that are taped to the skin were used to validate the models and good concurrent validity with an optoelectronic system was shown. To validate the novel sensor tights, a similar protocol was performed. Since the previous study was already performed with 11 participants, it was chosen to compare the previous results and the results with the new garment of 1 participant to validate the new product. In the current experiment, one male participant (age: 23 years, height: 1.89 m) was wearing the garment with integrated IMUs, and the measurements of the garment were compared with the optoelectronic system measurements. The sensor-to-segment calibration consists of two steps. First, the participant is asked to stand still for 5 s in a neutral upright pose to identify the longitudinal axis of each segment by using the direction of gravity. Thereafter, the participant performs three movements in the sagittal plane to determine the frontal axis of each segment; a rise of the right upper leg, a rise of the left upper leg, and a bow forward of the trunk. A gradient descent Madgwick algorithm is used to estimate the sensor orientation during the measurements (Madgwick et al., 2011).

The optoelectronic system used eight cameras (Vicon V5 cameras, Vicon Motion Systems Ltd., UK), and 20 reflective markers were placed at the lower body. More information about marker placement can be found in Appendix A. The motion capture area was 25 m^2^. Five types of football-specific movement were executed: an acceleration run, a run with a cutting movement, a run with a 180° turn, kicking a ball (preceded by a few steps) and a jump. Each movement was performed at three different intensities (50, 80, and 100% of maximum effort respectively). Each trial was repeated three times. A more detailed description of the protocol can be found in Wilmes et al. (2020). Data were processed in MATLAB (The MathWorks, Santa Clara, CA). Hip and knee joint angles and angular velocities were calculated with the data from the optoelectronic system, and with the data from the sensor tights. Thereafter, the root mean square differences (RMSDs) and coefficients of multiple correlation (CMCs) were calculated between the sensor tights and the optoelectronic system for all types of movements separately.

#### Field tests

Five male participants (recreational football players, age: 21.8 ± 1.3 years) were asked to wear the garment and to perform a high intensity football-specific training drill (Kelly et al., 2013) This training drill is aimed to replicate the physical movements and technical actions during match-play. The drill was performed at least two times for each participant. Next to the training drill, two participants performed an extra series of isolated football-specific movements at 50, 80, and 100% of maximum effort. These movements included an instep soccer kick, running, jumping vertically, running with the ball, and running sideways. After the field experiment, all participants were asked to fill in a short questionnaire about their experience with wearing the tights. Data were processed in MATLAB (The MathWorks, Santa Clara, CA). Differentiated signals and probability distributions of the decompressed accelerometer, gyroscope, and magnetometer data were calculated to check the completeness of the dataset, and functioning of the sensors. Furthermore, compression ratios for each type of movement were calculated, to test the performance of the data compression algorithm. The compression ratio (CR) is defined as:
(1)

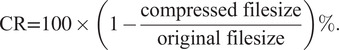



## Results and Discussion

This section describes the results and discussion of the validation study. First, a comparison of football-specific movements measured by the optoelectronic system and by the prototype is made. Secondly, the results of the recorded training sessions and user tests are discussed.

### Lab Validation

**Figure 3.**
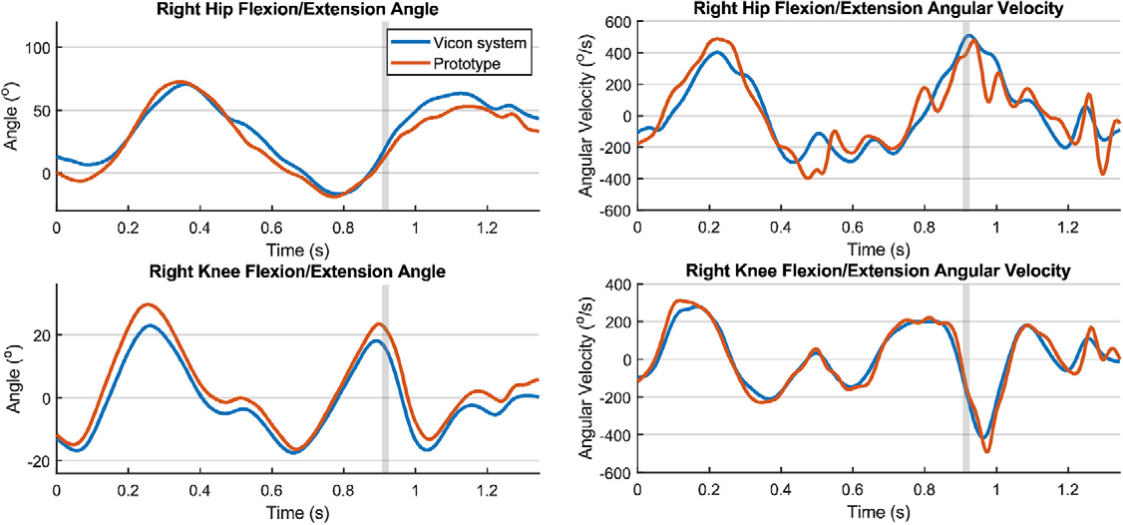
Joint angles and angular velocities of the right leg during a kick. The vertical gray line indicates the moment of ball contact.

**Figure 4.**
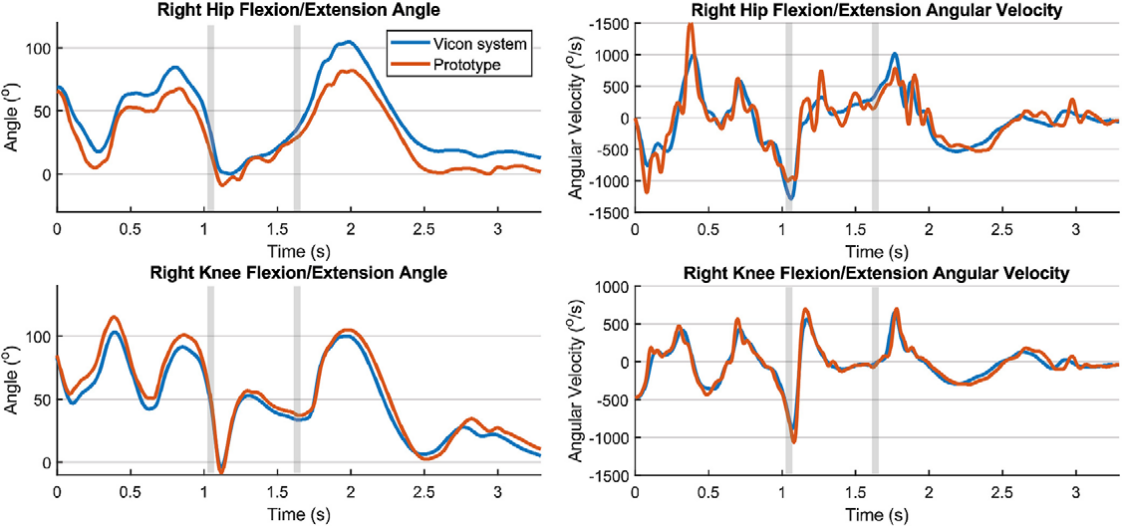
Joint angles and angular velocities of the right leg during a jump. The first vertical gray line indicates the time of push-off and the second indicates the landing.

One participant performed six types of football-specific movements, and these were tracked with the IMU system and the optoelectronic measurement system at the same time. To compare the IMU data with the optoelectronic measurement results, the raw IMU data were converted to joint angles and joint angular velocities. An example of the comparison of the joint angles and angular velocities of the right leg during a kick and a jump measured with the prototype and the optoelectronic system, is shown in Figures 3 and 4, respectively.

To evaluate the comparison, the RMSDs and coefficients of multiple correlation (CMCs) were calculated for each joint respectively. An overview of all RMSDs and CMCs at the different intensities and types of movement, is given in Appendix B. As shown in Table B1, RMSDs of the knee joint angles for all types of actions (each movement at a certain intensity) ranged between 14.11° and 2.98°, and CMCs between 0.911 and 0.997. For hip joint angles for all types of movement, RMSDs ranged between 18.46° and 7.07° and CMCs between 0.830 and 0.983. Errors in hip joint angles are likely to be larger than errors in knee joint angles, because the hip has a larger range of motion. RMSDs of joint angular velocities for all separated actions ranged between 168.2°/s and 59.1°/s for the hips and between 141.2°/s and 37.7°/s for the knees, respectively. CMCs ranged between 0.809 and 0.968 for the hip and between 0.979 and 0.9938 for the knee joint angular velocities.

In general, it was found that the RMSDs and CMCs for all types of movement are within the same range as in the previous measurements of 11 players with the taped IMUs (Wilmes et al., 2020). In some cases (e.g., Figure 4, upper right) it seems that the differences are related to stronger filtering (peak suppression) by the Vicon system, rather than measurement accuracy. Furthermore, the RMSDs and CMCs of joint angles found in the previous study by Wilmes et al. and in this study were comparable to the results from other studies. For example, Nüesch et al. (2017) measured RMSDs of 27.6° for the hip and 17.9° for the knee during jogging (~2.9 m/s), before they performed an offset correction. Additionally, Tadano et al. (2013) measured RMSDs of 9.0° for the hip and 7.1° for the knee during walking. Errors are expected to be higher for high intensity movements due to STAs and errors originating from the orientation filter. During this study, the participants performed higher intensity movements than reported in the other studies (e.g., acceleration runs with a mean running speed up to ~6.6 m/s), and the new IMU system still provides valid joint angle measurements (CMCs > 0.8) during these higher intensity movements. The advantage of the garment compared to the taped IMUs is that it is easier to use, comfortable to wear, and allows for longer-term monitoring studies during trainings and matches in the field.

#### Field tests

This section describes the results and discussion of the field tests. First, the technical validation results are discussed. Second, user experience insights are shown.

##### Technical validation

**Figure 5.**
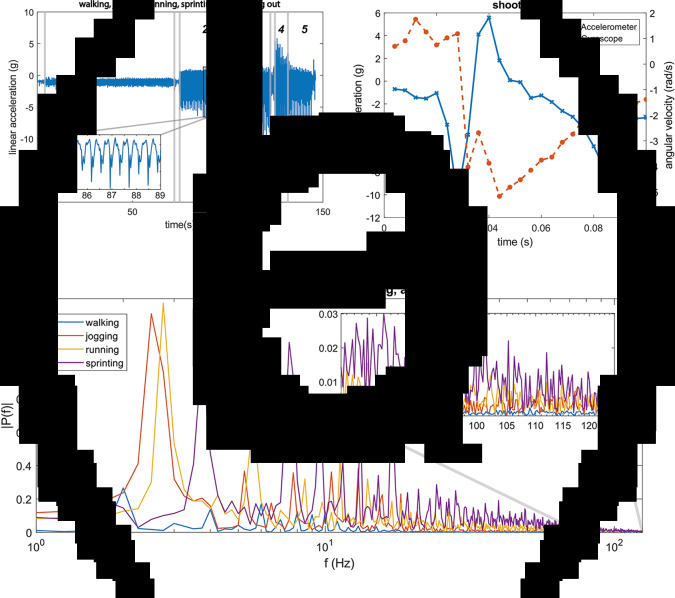
(a) Results from the left lower leg accelerometer (*x*-axis) of participant 1, during walking (1), jogging (2), running (3), sprinting (4), and running out (5). The spacings between each movement type indicate that the player is standing still or turning. (b) A recording of a shot. Results from the right lower leg accelerometer and gyroscope in a single direction. (c) Power spectrum analysis of accelerometer recordings (left lower leg, *x*-axis) at different running intensities (walking, jogging, running, and sprinting). For each running intensity, a recording of 1,000 samples (4 s) is used. A zoomed-in view of the range between 80 and 125 Hz is shown at the top right. The training session measurements showed that the accelerometers and gyroscopes worked properly and data transfer using the SPI protocol through the laced wiring appeared to be no problem. However, the magnetometers occasionally showed double readings. Most likely, this is caused by a not-updated register of the sensor itself. In future versions, the magnetometer can be replaced with a newer version, which may improve reliability of the readings.

Datasets of the training sessions of the five participants were checked for completeness by plotting the probability distributions of the differentiated signals and visually inspecting the sensor data. It was found that for one participant, malfunctioning of a connector resulted in data loss of one of the lower leg sensors. For the other measurements, the differentiated signals of the sensors showed a Gaussian distribution. Figure 5a shows typical accelerometer data of the left lower leg in a single direction for the accelerometer. They were recorded during execution of a series of isolated movements. The figure shows results at different running intensities.

Detailed analysis showed that the compression ratios varied with movement intensity as expected. Lower compression ratios were reached at higher movement intensities, whereas the lowest compression ratios were observed while sprinting (~24%). During walking the compression ratios were around 48%. As a comparison, Chiasson et al. (2020) were able to achieve compression ratios of ~16% of IMU data during walking in a recent study. It should be noted that their data were converted using the CR definition presented in the methods section, because they used another CR definition. The sample frequency of their dataset was 60 Hz, with a lower measurement range, which probably explains the large difference with our data. On top of that, the encoding part of the FELACS algorithm improves the representation of the quotient, most likely outperforming normal Golomb-rice encoding used by Chiasson et al. In this study, the average compression ratios of all sensors during the training session were between 43–45% for the different participants. In other words, the files of the training session data were 43–45% smaller than the original data set, which is beneficial for wireless data transfer in future prototypes. The algorithm for data compression consumes only 1.6% of the total power consumption extra power. These results are promising for wireless data transfer because the algorithm would markedly reduce the amount of data to be transferred at only a very small energetic cost.

At the start of this research, it was assumed that high sample rates (of 250 Hz) are required to prevent aliasing, and to ensure that the resolution is high enough to track all football-specific movements accurately. First, when inspecting the raw data of the recordings of the isolated movements, sudden large changes were observed during high intensity movements such as sprinting and kicking. Figure 5b shows the data of the gyroscope and accelerometer of the right lower leg during a shot. The recording comprises 25 samples. Due to the limited number of samples, no transient behavior was recorded during impact with the ball. The accelerometer measured a difference of almost 15 g within 12 ms. This indicates that maybe even higher sampling rates than 250 Hz could be beneficial for high intensity actions like shooting. In lower intensity movements walking, transient information and even noise were measured during a stride, so the sample rate is high enough for this type of movement.

Secondly, to analyse if the high sampling rate of 250 Hz is sufficient, a fast Fourier transform (FFT) was used to represent the data at different running intensities in the frequency domain. A total of 1,000 samples of each running intensity were used for a separate power spectrum analysis. The results are plotted in Figure 5c. The first peak in the power spectrum corresponds to the dominant movement frequency of the different activities. As can be seen from Figure 5c, for walking, jogging, running, and sprinting, the dominant frequencies increase from 2.0, 2.5, 2.75 to 3.75 Hz respectively. When zooming in at the last part of the graph, it can be seen that between 80 and 125 Hz the amplitude is higher for sprinting than for walking. A shock usually consists of a high magnitude main peak, associated with lower magnitude components at the higher frequency part of the spectrum. The high frequency components observed in Figure 5c are therefore attributed to these associated lower magnitude components. From the power spectrum analysis (Figure 5c) in combination with the abrupt changes in accelerometer and gyroscope data during shooting (Figure 5b) and sprinting, it can be concluded that this high sample rate adds information in case of high intensity movements. Further research is needed to find out if this extra information adds accuracy in deriving kinematics and estimating player load. Furthermore, a balance between high sample rates and practical implementation needs to be found. High sampling rates will increase the load on a wireless link in the future and it will increase energy use. Based on these considerations, a sampling frequency of 250 Hz is a justified choice. Future work may also include the use of adaptive sampling. For sprinting and shooting, the sample rates maybe even higher than 250 Hz, while the sample rates could be reduced for walking without losing accuracy.

##### User experience

To investigate user experience, each participant filled in a short questionnaire. Table 2 summarizes the results. Testing the prototype in the field provided insight not only in user experiences, but also in reliability of the prototype and efficiency and effectiveness of the movement monitoring system. User experience scores on comfort and freedom of movement were relatively high. For both attributes a rating of 4 was given on average (Likert 5-point scale). A tight fit of the tights is required for minimizing motion artifacts of the sensors. Four subjects experienced the tightness of the garment as a positive aspect. One subject, with the largest clothing size, experienced the garment as too tight. In the future, multiple sizes and shorts need to be developed to enhance comfort and freedom of movement for every player. In general, the subjects did not feel restricted in their movements by the electronics in the tights. However, one subject noted that the garment would seem too fragile for sliding’s. In future versions, connections will be made more robust to enable the players to make sliding’s. Furthermore, the thickness of the sensor modules will be reduced, and the use of a cushioning layer can be explored.

**Table 2.** Results of the user experience tests. Positive feedback is indicated with a (+) and negative feedback with a (−)

Subject		Size waist (cm)	Size inseam (cm)	Comfort (5-point Likert scale)	Freedom of movement (5-point Likert scale)	Qualitative feedback
1		79	86	4	5	+ Can be worn underneath football shorts − Garment feels very tight
2		76	81	3	2	+ Garment is tight − Not suitable for sliding’s
3		76	81	4	5	+ Very stretchable − Sensors seem fragile
4		79	81	3	4	+ Not too tight − Probably not suitable in summer
5		76	81	5	5	+ The same as ordinary tights − Feels hot during exercise
Mean (SD)		78 (2)	82 (2)	4 (1)	4 (1)	

When a novel prototype is developed and a wireless module is added to the tights, it would be beneficial when the athletes can activate the sensor tights themselves. Therefore, future work will include optimization of the user interface to start and stop measurements for use by athletes. Future work should also include better integration and miniaturization of the sensor nodes and central unit to enhance safe use and comfort. Furthermore, the connections with the sensor nodes need to be improved. During the tests, it was found that even after very short instants of disconnection, for example during dressing, the data collection is interrupted for this sensor. Since the connectors chosen were not designed for use in clothing, alternative solutions need to be explored. A very robust, yet not very sustainable solution, is to solder the wires to the PCB and encapsulate sensor nodes with an acrylate or silicone material. An alternative solution would be to develop a new type of connection, for example based on inductive coupling. In the current version of the garment, a waterproof textile layer was placed in the pockets between the electronics and the skin to protect against sweat. When the sensor nodes are encapsulated, the central unit casing is sealed, and an extra waterproof layer will be placed between the environment and the body, the system will also be resistant to rain. Lastly, although power consumption did not cause feasibility issues, it can be optimized to minimize the size of the battery. A smaller battery can enhance comfort and reduce safety issues. Power consumption can be reduced by optimizing the software and reducing power dissipation through the wiring.

## Conclusions

In this study, a fully functional prototype of a garment was developed for monitoring lower limb kinematics during every day, on-the-field training situations. Attention was paid to the aspects of unobtrusiveness, easiness of use, as well as the accuracy and reliability of the recorded signals. In a human performance lab, hip and knee joint angles and angular velocities recorded with the tights were compared with the same measurements obtained with an optoelectronic measurement system and good validity was shown. This demonstrates that the sensor tights can be used to accurately monitor lower limb kinematics. During the field tests, the tights scored high on ease-of-use, comfort, and freedom of movement, which allows the sensor garment to be used in field training sessions and matches, and this shows that the tights can be used for long term monitoring studies. In follow-up studies, it will be researched how the lower limb kinematics, measured by the garment, can be used to estimate individual player load on the lower limbs. Future work includes making a wireless connection to a dashboard for the coach and medical staff. This dashboard should show physical player load parameters. The ultimate goal is to relate these player load parameters to the prevalence of hamstring injuries, so that the tights can be used to prevent hamstring injuries in the future.

## Figures and Tables

**Figure A1.**
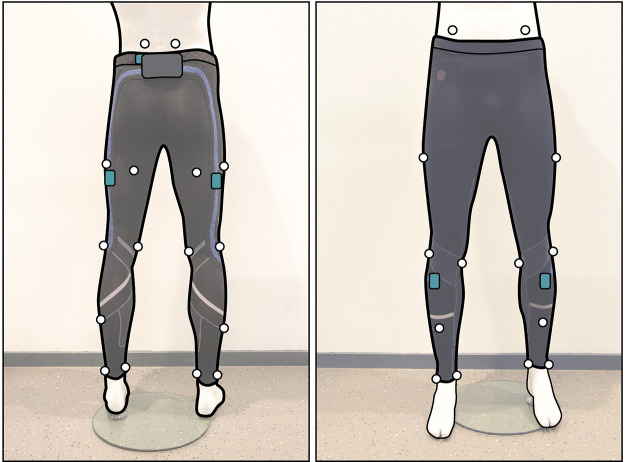
The setup for the validation experiment from posterior (left) and anterior (right) view: the IMUs are highlighted in blue. The Vicon markers (white dots) are placed at the following anatomical locations: At the posterior and anterior superior iliac spines, Halfway the length of hip and knee on the lateral and posterior sides of the thighs, at the medial and lateral femoral epicondyles, halfway the length of knee and ankle on the lateral and anterior sides of the shanks, and at the medial and lateral malleoli.

## Data Availability

The datasets used in this study are available from the corresponding author upon request.

## B. Appendix B

### B.1. Results of the Lab Validation Study

**Table B1.** Root mean square differences between the optoelectronic measurement system and the prototype for the different movement types at different intensity levels

Movement	Left hip angle (º)	Right hip angle (º)	Left knee angle (º)	Right knee angle (º)	Left hip angular velocity (º/s)	Right hip angular velocity (º/s)	Left knee angular velocity (º/s)	Right knee angular velocity (º/s)
Run 70%	8.88	10.55	7.47	4.12	87.79	75.85	68.71	81.73
Run 85%	7.63	11.14	7.03	5.49	99.05	91.10	80.76	90.08
Run 100%	10.71	8.72	2.98	8.88	168.24	161.05	80.19	141.24
Turn 70%	16.80	7.07	6.76	13.27	65.91	62.22	77.04	70.56
Turn 85%	14.83	7.77	6.46	14.11	64.49	68.48	73.90	77.29
Turn 100%	17.08	9.52	8.07	13.33	61.48	87.62	58.13	91.83
Cut 70%	12.09	12.75	6.14	6.79	138.33	98.78	65.68	58.56
Cut 85%	11.77	12.93	5.68	6.64	84.21	89.51	81.20	53.10
Cut 100%	11.33	10.71	6.18	9.86	95.03	80.18	80.33	52.40
Jump 70%	14.23	10.05	4.61	8.54	65.85	59.08	37.69	45.27
Jump 85%	14.20	9.39	4.88	8.55	61.89	66.90	57.32	73.84
Jump 100%	15.35	13.62	4.58	7.38	61.08	71.65	78.29	64.81
Kick 70%	12.75	10.70	3.91	7.34	61.24	81.63	68.66	68.10
Kick 85%	14.43	9.51	6.05	8.05	76.87	87.80	84.97	77.71
Kick 100%	18.46	8.08	11.63	11.20	93.01	103.10	79.26	96.25
Mean	13.37	10.17	6.16	8.90	85.63	85.66	71.48	76.18

**Table B2.** Coefficients of multiple correlation between the optoelectronic measurement system and the prototype for the different movement types at different intensity levels

Movement	Left hip angle	Right hip angle	Left knee angle	Right knee angle	Left hip angular velocity	Right hip angular velocity	Left knee angular velocity	Right knee angular velocity
Run 70%	0.952	0.952	0.990	0.996	0.948	0.969	0.993	0.989
Run 85%	0.972	0.957	0.992	0.995	0.947	0.968	0.993	0.990
Run 100%	0.966	0.983	0.997	0.989	0.913	0.946	0.994	0.989
Turn 70%	0.853	0.956	0.982	0.930	0.933	0.965	0.978	0.989
Turn 85%	0.872	0.957	0.986	0.911	0.916	0.963	0.983	0.984
Turn 100%	0.855	0.948	0.977	0.936	0.945	0.953	0.988	0.982
Cut 70%	0.855	0.892	0.983	0.979	0.809	0.936	0.989	0.990
Cut 85%	0.788	0.780	0.979	0.978	0.907	0.945	0.983	0.994
Cut 100%	0.757	0.868	0.979	0.951	0.906	0.962	0.983	0.992
Jump 70%	0.940	0.971	0.996	0.987	0.955	0.964	0.992	0.989
Jump 85%	0.932	0.968	0.994	0.981	0.956	0.955	0.985	0.979
Jump 100%	0.920	0.942	0.995	0.986	0.961	0.953	0.979	0.983
Kick 70%	0.628	0.896	0.992	0.952	0.927	0.946	0.987	0.979
Kick 85%	0.830	0.944	0.985	0.972	0.938	0.938	0.982	0.987
Kick 100%	0.839	0.977	0.948	0.966	0.945	0.949	0.984	0.987
Mean	0.864	0.933	0.985	0.967	0.927	0.954	0.986	0.987
